# Supplementary material for: Causal Relationship between Aging and Anorexia Nervosa: A White-Matter-Microstructure-Mediated Mendelian Randomization Analysis
Source: Biomedicines. 2024 Aug 16;12(8):1874. doi: 10.3390/biomedicines12081874 (PMC11351342; doi:10.3390/biomedicines12081874)
Supplement: Supplementary file 1 [file biomedicines-12-01874-s001.zip › Supplementary Figure/Figure S9/Leaveoneout plot of MR analysis for ubm-b-1493 to AN.pdf]

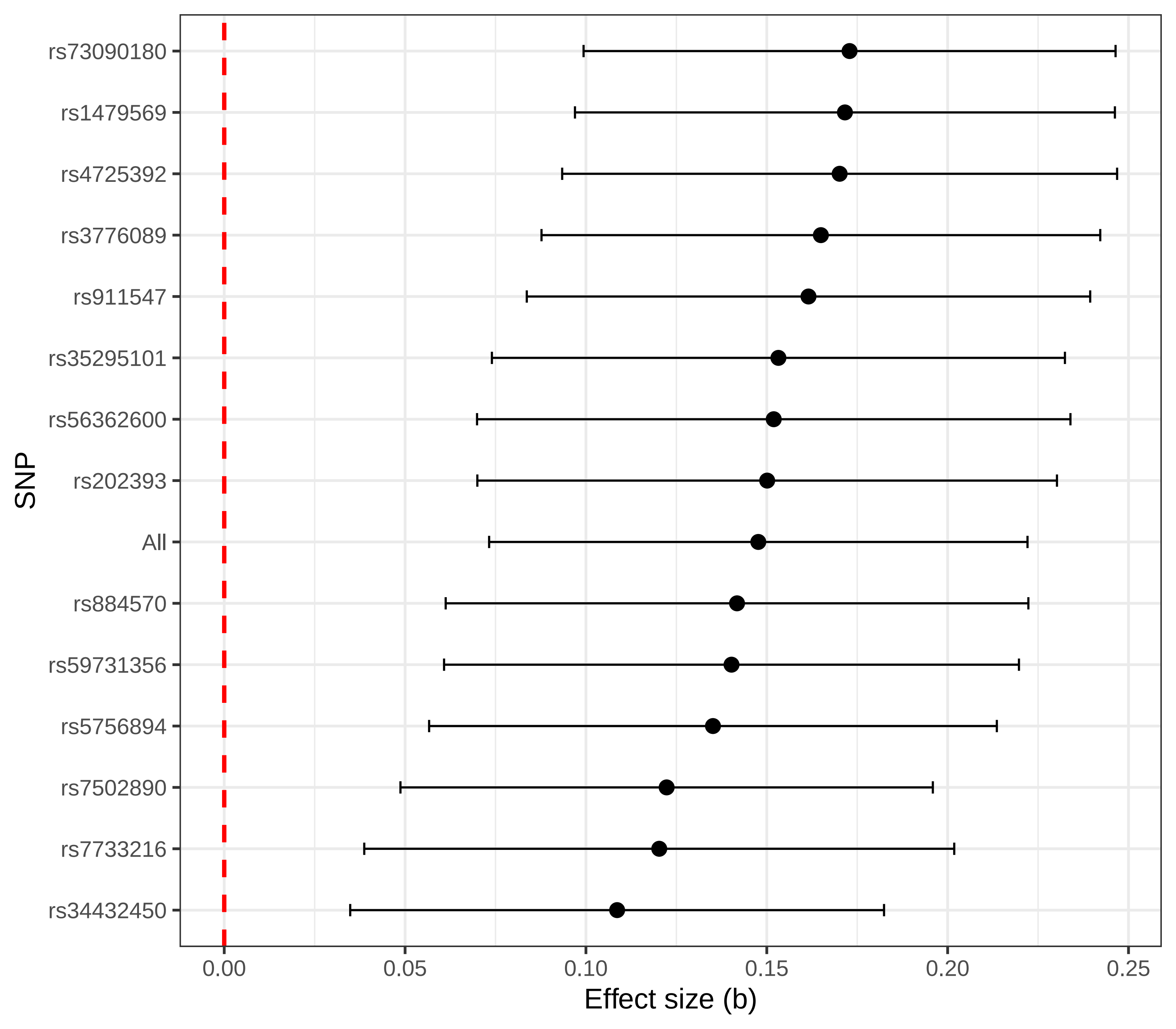

rs73090180
rs1479569
rs4725392
rs3776089
rs911547
rs35295101
rs56362600
rs202393
All
rs884570
rs59731356
rs5756894
rs7502890
rs7733216
rs34432450
SNP
0.00
0.05
0.10
0.15
0.20
0.25
Effect size (b)
